# Association between the HLA-B*1502 gene and mild maculopapular exanthema induced by antiepileptic drugs in Northwest China

**DOI:** 10.1186/s12883-021-02363-w

**Published:** 2021-09-06

**Authors:** Nilupaer Shafeng, Deng-feng Han, Yun-fang Ma, Rena Abudusalamu, Binuer Ayitimuhan

**Affiliations:** 1 Department of Neurology, First Affiliated Hospital of Xinjiang Medical University, 830000 Urumqi, Xinjiang Province China; 2 Department of Clinical Pharmacy, First Affiliated Hospital of Xinjiang Medical University, Xinjiang Province 830000 Urumqi, China

**Keywords:** maculopapular exanthema, HLA-B*1502, antiepileptic drugs, HLA genotype

## Abstract

**Background:**

The relationship between the HLA-B*1502 gene and maculopapular exanthema (MPE) induced by antiepileptic drugs (AEDs) has not yet been elucidated. In this study, we investigated the association between AED-induced MPE (AED-MPE) and the HLA-B*1502 gene in patients in Northwest China.

**Methods:**

We enrolled 165 subjects including nine patients with AED-MPE and 156 AED-tolerant patients as controls. HLA-B*1502 gene polymorphism was detected using digital fluorescence molecular hybridization (DFMH). The results of HLA genotyping were expressed as positive or negative for the HLA-B*1502 allele. An analysis of AED-MPE risk factors was performed using binary logistic regression, and differences in genotype frequencies between groups were assessed with the continuity correction chi-square test.

**Results:**

We found that the HLA-B*1502 gene was a risk factor for AED-MPE (*P* = 0.028). The incidence of MPE induced by the two types of AEDs was different, and the incidence of aromatic AEDs use was higher that of non-aromatic AEDs use (*P* = 0.025). The comparison of the gene frequencies of the HLA-B*1502 allele between the two groups taking aromatic AEDs was also statistically significant (*P* = 0.045). However, there were no significant differences in terms of age, gender, ethnicity, or region in patients with MPE induced by AEDs. In addition, no association between the HLA-B1502 allele and CBZ- or OXC-induced MPE was found.

**Conclusions:**

In northwestern China, the HLA-B*1502 allele was associated with aromatic AED-MPE. Since MPE can develop into Stevens–Johnson syndrome (SJS) or toxic epidermal necrolysis (TEN), the HLA-B*1502 gene should be evaluated before administering AEDs.

## Background

Epilepsy (EP) is a disease of the nervous system characterized by epileptic seizures (EPSs). An EPS is a transient clinical symptom caused by abnormal synchronous discharge and excessive activity of brain neurons [1]. At present, there are approximately 10 million people living with EP in China; the prevalence rate is approximately 7 %, with an annual increase of 450,000 people, and the condition seriously affects patients’ lives [2]. At present, doctors commonly use common AEDs including phenytoin (PHT), phenobarbital (PB), carbamazepine (CBZ), oxcarbazepine (OXC), valproate (VPA), and lamotrigine (LTG) as well as a new generation of AEDs, such as levetiracetam (LEV) and lacosamide (LCM). However, because of the uncertain efficacy and high prices of these new AEDs, traditional aromatic AEDs are still the first-line treatment for epilepsy. Different individuals respond differently to antiepileptic medications. Of the different responses, the most common and dangerous are adverse drug reactions (ADRs), also known as type-B ADRs [3]. Depending on the severity of the drug eruption, the main symptoms are maculopapular exanthema (MPE) and severe cutaneous adverse reactions (SCARs) with potentially life-threatening consequences, including drug reactions with eosinophilia and systemic symptoms (DRESS), Stevens–Johnson syndrome (SJS), and toxic epidermal necrolysis (TEN); the mortality rate for the latter two diseases is as high as 30 %, and the fatality rate is very high [4, 5]. Recent studies have reported a strong correlation between HLA-B*1502 and CBZ-induced SJS/TEN in Asian populations [6], and in 2008, the US Food and Drug Administration (US FDA) also recommended genetic screening of the HLA-B*1502 allele prior to initiation of CBZ therapy in Asians [7]. As a derivative of CBZ, OXC is relatively safe to use, whereas some studies have shown that OXC-induced cutaneous adverse drug reactions (CADRs) are related to HLA-B*1502, of which MPE is the most common [8]. However, some studies have found no correlation between the HLA-B*1502 gene and OXC-induced MPE (OXC-MPE) in northern and southern China [9, 10]. This study was conducted because these current results are controversial and because no studies on the HLA-B*1502 gene and AED-induced MPE (AED-MPE) have been previously conducted in northwest China.

## Methods

### Patients

This was a case–control study. Patients indigenous to mainland China were from towns of Xinjiang Province in northwestern China. The research protocols were approved by the Ethics Committee of the First Affiliated Hospital of Xinjiang Medical University, and we obtained written clinical informed consent for patients with EP. A total of 165 epileptic patients taking a single AED were included in the outpatient department and in-patient departments of neurology and neurosurgery of the First Affiliated Hospital of Xinjiang Medical University from January 2018 to May 2020. Among them, nine patients who met the diagnostic criteria for AED-MPE were identified. MPE was defined as erythematous exanthema without blistering or pustulation. No cases of SJS/TEN or DRESS were collected in this study. All the patients were diagnosed and treated by the treating epileptologist and dermatologists. We enrolled 156 patients with AED who had more than three months of AED and no CADRs as the tolerant group (AED-tolerant group). All patients were from three ethnic groups in China: Han, Uygur, and Kazak. Data were collected based on patients’ age, sex, ethnicity, region, and AED types.

### Methods

Genomic DNA was extracted from 2 to 3 ml of patients’ peripheral venous blood. The samples were preserved with nucleic acid purifying reagent (Yao Jinbao) and were analyzed by a general reagent kit for sequencing reactions (Yao Jinfen). HLA-B*1502 genotypes were analyzed by digital fluorescence molecular hybridization (DFMH) (TL998A fluorescence detector; Xi’an Tianlong Science and Technology Co., Ltd., Xi’an, China). Genotyping results are presented as positive or negative.

### Statistical analysis

Analysis of AED-MPE-related risk factors was performed by binary logistic regression. The continuity correction chi-square test was performed to analyze the association between AED-MPE and HLA-B*1502 allele. P values less than 0.05 (two-sided) were considered statistically significant. All data were analyzed using SPSS 22.0 (IBM, Armonk, NY, USA).

## Results

Of the 165 patients with EP treated with AEDs (*N* = 165), nine patients who had been diagnosed with AED-MPE were treated with aromatic AEDs (CBZ/OXC). The nine patients included six males and three females: five Han, three Uygur, and one Kazak. Eight patients were HLA-B*1502-gene positive (all mutant heterozygotes), and one patient was negative for HLA-B*1502. Table 1 lists the clinical features and HLA-B*1502 gene results of nine patients with AED-MPE (Table 1). In the AED-tolerant group, there were 100 males and 56 females: 61 Han, 69 Uygur, and 26 Kazak. Seventy-two patients were positive for HLA-B*1502 (four mutant homozygotes and 68 mutant heterozygotes), and 84 patients were negative for HLA-B*1502. After eliminating confounding factors, the results of binary logistic regression showed that the HLA-B*1502 gene was a risk factor for AED-MPE (*P* = 0.028; OR = 11.028; 95 % CI, 1.296–93.818), which was statistically significant. These results indicated that the risk of HLA-B*1502-gene-positive patients developing MPE after taking AEDs was 11.028 times that of gene-negative patients. However, age, gender, ethnicity, and geographical factors were not related (Table 2).

**Table 1:** Characteristics of patients with MPE induced by AEDs

ID	sex (F/M)	ethnicity	disease	drug	HLA-B*1502gene
1	M	Han	EP	CBZ	-
2	M	Han	EP	CBZ	+
3	M	Uygur	EP	CBZ	+
4	M	Uygur	EP	OXC	+
5	F	Han	EP	OXC	+
6	M	Han	EP	OXC	+
7	F	Uygur	EP	CBZ	+
8	M	Kazak	EP	CBZ	+
9	F	Han	EP	OXC	+

F: female; M: male; EP: epilepsy; CBZ: carbamazepine; OXC: oxcarbazepine

**Table 2:** Binary logistic regression analysis of different factors contributing to AED-MPE^1^

variable	B^2^	P^2^ value		Crude OR,95 % CI	Adjusted OR,95 % CI	
age	0.013	0.493		1.014(0.976–1.053)	1.013(0.976–1.052)	
sex	-0.078	0.919		1.120(0.270–4.652)	0.925(0.206–4.158)	
ethnicity						
Han	-	0.664		-	-	
Uygur	-0.556	0.559		0.530(0.122–2.312)	0.573(0.089–3.697)	
Kazak	-0.888	0.443		0.469(0.052–4.216)	0.412(0.043–3.979)	
HLA-B*1502 gene^3^	2.400	0.028^*^		9.333(1.140-76.412)	11.028(1.296–93.818)	
region^4^	-0.317	0.737		1.152(0.277–4.782)	0.728(0.114–4.650)	
constant	-4.625	0.001		-	0.010	

^1^AED-MPE: antiepileptic drug-induced maculopapular exanthema

^2^B: the regression coefficient after eliminating confounding factors.P:P value after eliminating confounding factors

^3^HLA-B*1502 gene: positive

^4^ Region: Northern Xinjiang region of China, Southern Xinjiang region of China

Among the 165 patients taking AEDs, 97 were taking aromatic AEDs, and 68 were taking non-aromatic AEDs. Comparison of the AED-MPE and AED-tolerant groups showed that the relationship between the use of aromatic AEDs and induced MPE was statistically significant (*P* = 0.025). Among patients taking aromatic AEDs, the frequency of the HLA-B*1502 gene was statistically significantly higher in the AED-MPE group than in the AED-tolerant group (*P* = 0.045; OR = 8.76; 95 % CI, 1.05–73.04) (Table 3).

**Table 3:** Comparison of AEDs types and HLA-B*1502 gene frequencies between AED-MPE^1^ and AED-tolerant groups

	Drug2		Aromatic antiepileptic drugsHLA-B*1502 allele^3^		Gene frequency^3^(%)	P value^2^	P value^3^
	Aromatic	Non-Aromatic	+^4^	-^4^			
AED-MPE	9	0	8	1	88.9	0.025*	0.045^*^
AED-tolerant	88	68	42	46	47.7		
Total	97	68	50	47	51.5		

^1^AED-MPE: antiepileptic drug-induced maculopapular exanthema

^2^AED-MPE compared with AED-tolerant group in different drug types: x^2^ = 4.995

^3^ AED-MPE compared with AED-tolerant group in aromatic antiepileptic drugs HLA-B*1502 allele: x^2^ = 4.013

^4^+: positive; −: negative

*P < 0.05(two-sided) was statistically significant

**Fig. 1:**
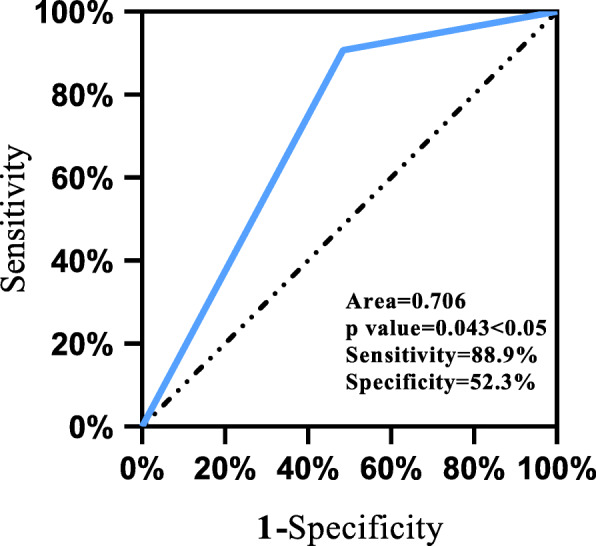
ROC curve of HLA-B*1502 gene screening

The sensitivity of detection of the HLA-B*1502 gene in the aromatic AED-MPE and AED-tolerant group was 88.9 % (8/9), the specificity was 52.3 % (46/88), the positive predictive value was 16 % (8/50), and the negative predictive value was 97.9 % (46/47). The sensitivity and negative predictive value were high, and the specificity was moderate (Fig. 1).

In the AED-MPE group, there were five cases of CBZ-induced MPE (CBZ-MPE): four patients were positive for the HLA-B*1502 gene, and one patient was negative for the HLA-B*1502 gene. Compared with patients taking CBZ in the tolerant group (19/48), there was no significant correlation between the two groups (*P* = 0.207). Similarly, there were four patients with OXC-MPE, and all four were gene-positive. Compared with patients taking OXC in the tolerant group (23/40), the results showed no statistically significant differences (*P* = 0.260) (Table 4). The collated data revealed that one patient in the AED-MPE group was negative for the HLA-B*1502 gene, and four patients in the tolerant group were homozygous for the mutant HLA-B*1502 gene.

**Table 4:** Relationship between the HLA-B*1502 gene and aromatic AED-MPE^1^

Type	*N* = 97	HLA-B*1502	Non HLA-B*1502	χ^2^	P Value
AED-MPE					
CBZ^2^	5	4	1	1.591	0.207
OXC^3^	4	4	0	1.268	0.260
Tolerant group					
CBZ	48	19	29	-	-
OXC	40	23	17	-	-

^1^AED-MPE: antiepileptic drug-induced maculopapular exanthema

^2^ CBZ-MPE compared with CBZ-tolerant control

^3^ OXC-MPE compared with OXC-tolerant control

## Discussion

That AEDs induce CADRs is a global issue of concern. Drug-induced CADRs are mainly due to delayed drug hypersensitivity mediated by T-cell immunity, which usually appears several days or even weeks after drug administration. The clinical manifestations are mainly MPE with mild symptoms, and SCARs include SJS, TEN, DRESS, drug-induced hypersensitivity syndrome (DIHS), and acute systemic rash impetigo (AGEP) [3, 11]. The mechanism underlying AED-induced CADRs is currently considered to be one of the following: hapten theory, pi concept, changed peptide library model, and changed T-cell receptor (TCR) library model [12]. However, the specific pathogenesis is more complicated and is still not fully understood; further research is required. At present, most studies have confirmed that HLA alleles are significantly associated with CADRs. In East Asia, the HLA-B*1502 allele is strongly correlated with CBZ-induced SJS/TEN, especially in the Chinese Han population [6]. Similar reports have confirmed this in Asian countries such as Malaysia [13, 14], Indonesia [15], and Thailand [16, 17], but it is rare in Europe, Japan, South Korea, and other regions. Therefore, in 2008, the US FDA recommended HLA-B*1502 genetic screening for all Asian patients before starting CBZ treatment [7].

In this study, binary logistic regression was used to analyze factors related to MPE induced by AEDs after eliminating confounding factors. It was found that age, gender, ethnicity, and region were not statistically significant, but HLA-B*1502 gene type was related to MPE induced by AEDs(*P* = 0.028; OR = 11.028; 95 % CI, 1.296–93.818), indicating that the HLA-B*1502 gene-positive is a risk factor for MPE. When the different types of enrolled AEDs (aromatic or non-aromatic) were compared, aromatic AEDs were found to be related to MPE (*P* = 0.025), which was consistent with the findings of a previous report that showed that AEDs with aromatic ring structures were associated with an increased incidence of skin adverse reactions [18]. However, this result did not clarify whether the HLA-B*1502 gene is related to MPE induced by aromatic AEDs. Therefore, after testing the HLA-B*1502 gene status of patients who using aromatic AEDs, eight patients in the AED-MPE group were found to be positive; one patient was negative. The positivity rate was therefore 88.9 % (8/9). In the tolerant group, there were 42 cases of HLA-B*1502 gene positivity, and 46 patients were negative for HLA-B*1502; the positivity rate was 47.7 % (42/88). A continuity correction chi-square analysis indicated that the HLA-B*1502 gene and aromatic AED-MPE were statistically significantly related (*P* = 0.045; OR = 8.76; 95 % CI, 1.05–73.04), indicating that HLA-B*1502 gene positivity was associated with MPE induced by aromatic AEDs. The OR > 1 indicates that HLA-B*1502 gene positivity is a risk factor for patients taking aromatic AEDs. Furthermore, we found that the sensitivity and negative predictive value of HLA-B*1502 allele detection for MPE induced by aromatic AEDs were high, and the specificity was moderate, indicating that when the HLA-B*1502 gene is negative, the chance of developing AED-MPE is small, providing more certainty for doctors formulating individualized antiepileptic treatment plans. In practical applications, a method with a high sensitivity and low price can be selected to conduct preliminary diagnosis and evaluation of patients. The prevalence of HLA-B*1502 gene-positive patients is higher than that of gene-negative patients to develop ARDs, thus, doctors must determine whether more accurate diagnostic tests can be used to confirm their diagnosis when analyzing patients’ diagnostic test results.

In the nine patients with AED-MPE, who had all taken aromatic AEDs (CBZ, 9.4 % (5/53); OXC, 9 % (4/44)), we found that when the CBZ-MPE/CBZ-tolerant group and OXC-MPE/OXC-tolerant group were compared in terms of the HLA-B*1502 allele, CBZ-MPE (*P* = 0.207) and OXC-MPE (*P* = 0.260) were not associated with HLA-B*1502 gene (*P* > 0.05). This finding was consistent with the results of a domestic study on the Han population in northern and southern China [9, 10, 19, 20]. Furthermore, a study in Thailand also found that MPE occurred in five of 81 patients taking CBZ, but there was no correlation between HLA-B*1502 gene and the tolerant group (*P* = 1.14; OR = 1.21; 95 % CI, 0.21–6.99) [21]. However, another study of the Chinese Han population showed that compared with a normal control group, OXC-MPE was significantly associated with HLA-B*1502 gene (*P* = 0.011; OR = 8.8; 95 % CI, 1.853–41.790). Although not statistically significant compared with the tolerant group, the prevalence of the HLA-B*1502 gene was higher in the OXC-MPE group than in the OXC-tolerant group. The HLA-B*1502 allele may increase the genetic risk of OXC-MPE in the Chinese Han population [8]. Our study was insufficient to draw conclusions because there was no normal control group. We will continue to expand the sample size and include a normal control group in future studies. Another study in Thailand also proposed that the HLA-B*1502 gene and CBZ-MPE were correlated (*P* = 7.35*10^− 12^; OR = 19.13; 95 % CI, 7.94–46.09) [17]. These two differences may be related to the different study populations, and the volume of data needs to be expanded to obtain additional statistics.

According to previous studies, the HLA-B*1502 gene has been confirmed to be significantly associated with CBZ-induced SJS/TEN [3]. Recently, a study from Taiwan also reported a significant association between HLA-B*1502 gene and SJS/TEN induced by CBZ (*P* = 1.6*10^− 44^; OR = 97.6; 95 % CI, 42.0–226.8) [22]. As a derivative of CBZ, OXC has relatively few side effects and high safety; therefore, it has been used as a first-line AED to replace CBZ. However, some studies have found that SJS/TEN also occurs in patients taking OXC. In a study of SJS/TEN induced by OXC in Asian populations (Chinese Han and Thailand), it was found to be significantly correlated with the HLA-B*1502 gene (*P* = 1.87*10^− 10^; OR = 27.90; 95 % CI, 7.84–99.23) [23]. MPE is one of the milder ADRs and is more common with AEDs. However, if MPE is not treated, it can further develop into SJS/TEN and pose a risk to life. Therefore, the 2017 Clinical Pharmacogenetics Implementation Consortium (CPIC) guidelines clearly recommended that before the first use of CBZ or OXC, the HLA-B*1502 gene status should be tested [24], and positive patients should discontinue CBZ or OXC and be given other AEDs. Therefore, it is still necessary to identify other relevant risk factors for the induction of MPE by AEDs.

In our case–control study, we found one case of HLA-B*1502 gene-negative AED-MPE: the patient was a 60-year-old male with a history of “viral encephalitis” in May 2018. Loss-of-consciousness and limb twitching occurred for the first time on October 31, 2018, mainly manifested as unresponsiveness, involuntary twitching of the limbs, prominent on the right side, clenched teeth, and upturned eyes; no nausea, vomiting, or incontinence was observed. The symptoms were relieved after sedation, but he had another seizure in January 2019 with head deflection to the right, twitching of the right upper extremities, salivation, clenched teeth, and upturned eyes; no incontinence or tongue biting was observed, and the seizure lasted approximately five minutes. After waking, he could not recall what had happened. Through the patient’s 24-hour electroencephalogram (EEG) results showed that there were no abnormalities. In view of the patient’s medical history over more than 24 h, there were two seizures without any obvious cause. The patient met the diagnostic criteria for EP, was diagnosed with EP, and was given 200 mg of CBZ orally twice a day. The patient developed a pale red rash surrounded by skin with redness after taking the drug for three months. The clinical manifestations were mainly hard red-colored nodules that were elevated above the surrounding skin, and the patient felt pain when these nodules were pressed. The rash first appeared on the neck and then quickly spread out on the limbs. Because the rash was occasionally itchy, he went to our hospital, where he was diagnosed with MPE after consultation with a neurologist, an EP physician, and a dermatologist. CBZ treatment was discontinued, and the symptoms resolved after anti-allergic treatment. For EP, it was recommended that the patient’s treatment be adjusted to 500 mg of LEV orally twice a day. The last seizure occurred in May 2019, of which the clinical manifestations were the same as the previous two seizures. The HLA-B*1502 genetic test was negative. There were no adverse skin reactions during the period of LEV treatment, and the patient’s seizures have been well-controlled to date. The patient has been seizure-free for nearly two years. Furthermore, among the patients we enrolled were four patients in the AED-tolerant group who were homozygous for the HLA-B*1502 gene mutation.

We considered that in addition to the HLA-B*1502 gene, there may be other HLA phenotypic genes that are associated with AED-MPE. At present, researchers believe that HLA-A*3101 is strongly correlated with AED-MPE [20, 25] and that HLA-A*3101 positivity is associated with MPE and DRESS induced by CBZ [26], while HLA-B*1502 positivity is more significant associated with CBZ induced SJS /TEN. Through peptide analysis and proteomic testing, Simper et al. [27] found that CBZ-induced drug hypersensitivities caused by HLA-A*3101 and HLA-B*1502 are two different diseases, HLA-A*3101-positive patients after taking CBZ,the drug binds to the autologous peptide of the HLA gene, which changes the high-binding peptide and simultaneously presents the low-binding peptide, thereby reducing the immune response and severity of MPE. In HLA-B*1502-positive patients, the binding of CBZ and the CBZ derivative EPX to the F pocket is related, which is mainly manifested as changes in the high-binding peptides. These changes will lead to a strong T-cell response and cause the occurrence of SJS/TEN. There are differences between the HLA-B*1502 gene distribution and that of the HLA-A*3101 gene, which is distributed all over the world [28], and studies have suggested that the HLA-A*3101 gene is relatively important for the induction of adverse skin reactions by AEDs. Furthermore, other studies have found that in addition to the HLA-B*1502 and HLA-A*3101 genes, HLA-A*2402, HLA-B*5801, HLA-B*3802, HLA-B*4002, DRB1*0403, HLA-B*1302, HLA-A*0201, and HLA-DRB1*1405 are associated with CBZ-MPE or OXC-MPE [9, 10, 17, 19, 29, 30]. In addition to different genes, Yang et al. [31]found an increase in the expression of cytotoxic cytokines in the serum and tissue of patients with CADRs in their study of the immune mechanism of adverse skin reactions in the Chinese Han population, suggesting that drug-induced adverse skin reactions and cytotoxic cytokines are related. Ko et al. [32] extracted the TCR from the serum of patients with SJS/TEN taking CBZ and found that in the patients positive for HLA-B*1502, the TCR clonal type VB-11-ISGSY was present, while in the CBZ-tolerant group, this clonal type was not found, indicating that the VB-11-ISGSY clonal type is related to the induction of SJS/TEN. We hypothesized that the reason why patients positive for the HLA-B*1502 gene in the aromatic AED-tolerant group did not have ADRs in this study may be related to this finding. The current mechanism relating aromatic AED-MPE and HLA genes is still unclear; thus, future research should continue to explore the mechanistic relationships between CBZ-MPE and OXC-MPE and HLA alleles. Since this was a small-sample study, our results may only be an incidental discovery and need to be further confirmed in a larger study. The sample size should be expanded to obtain more statistically relevant information.

The kit used in our case–control study was specifically designed to detect the presence of the HLA-B*1502 gene; therefore, other related genes could not be detected. It is necessary to expand the sample size further while detecting additional HLA-A and HLA-B genotypes, screening for AED-MPE-related genotypes, and providing essential references for clinical AED treatment programs.

## Conclusions

In summary, we found that the HLA-B*1502 allele was associated with aromatic AED-MPE in patients in northwestern China. Furthermore, no association was found between AED-MPE and age, gender, ethnicity, or region in northwestern Chinese population. There was also no significant association between the HLA-B*1502 allele and CBZ-MPE or OXC-MPE. The sample size of this study was small, and it should be expanded for further research. Although the HLA-B*1502 allele detection had high sensitivity and medium specificity in this study, the higher the sensitivity is, the more new cases can be identified, leading to lower missed diagnosis rates. Higher survival rates will be achieved by early detection, diagnosis, and treatment. We recommend that screening for the HLA-B*1502 gene be performed before administering CBZ or OXC.

## Data Availability

The datasets used and/or analysed during the current study available from the corresponding author on reasonable request.

## Abbreviations

MPE: maculopapular exanthema
AEDs: antiepileptic drugs
DFMH: digital fluorescence molecular hybridization
CBZ: carbamazepine
OXC: oxcarbazepine
SJS: Stevens–Johnson syndrome
TEN: toxic epidermal necrolysis
DRESS: drug reaction with eosinophilia and systemic symptoms
ADRs: adverse drug reactions
SCARs: severe cutaneous adverse reactions
CADRs: cutaneous adverse drug reactions
US FDA: United States Food and Drug Administration
TCR: T cell receptor
